# Vaccination and Omicron BA.1/BA.2 Convalescence Enhance Systemic but Not Mucosal Immunity against BA.4/5

**DOI:** 10.1128/spectrum.05163-22

**Published:** 2023-04-26

**Authors:** Gabriel Diem, Michael Jäger, Stefanie Dichtl, Angelika Bauer, Cornelia Lass-Flörl, Markus Reindl, Doris Wilflingseder, Wilfried Posch

**Affiliations:** a Institute of Hygiene and Medical Microbiology, Medical University of Innsbruck, Innsbruck, Austria; b Clinical Department of Neurology, Medical University of Innsbruck, Innsbruck, Austria; University of São Paulo

**Keywords:** COVID-19 vaccines, neutralizing antibodies, Omicron BA.4/5, SARS-CoV-2, systemic/mucosal immunity, adaptive immunity

## Abstract

Rising breakthrough infections with severe acute respiratory syndrome coronavirus 2 (SARS-CoV-2) Omicron BA.4/5 led to the performance of various studies investigating systemic immunity and neutralizing antibodies in sera, but mucosal immunity remains understudied. In this cohort study, the humoral immune responses, including immunoglobulin levels and the presence of virus-neutralizing antibodies, of 92 vaccinated and/or BA.1/BA.2 convalescent individuals were investigated. Cohorts received two doses of ChAdOx1, BNT162b2, or mRNA-1273 and subsequent booster vaccination with either BNT162b2 or mRNA-1273, following BA.1/BA.2 infection. In addition, vaccinated and nonconvalescent or unvaccinated and BA.1 convalescent individuals were studied. Serum and saliva samples were used to determine SARS-CoV-2 spike-specific IgG and IgA titers and neutralizing activity against replication-competent SARS-CoV-2 wild-type virus and the Omicron BA.4/5 variant. Vaccinated/convalescent cohorts demonstrated strongest neutralization against BA.4/5, with 50% neutralization titer (NT_50_) values reaching 174.2; however, neutralization was reduced up to 11-fold, compared to wild-type virus. Both BA.1 convalescent and vaccinated nonconvalescent cohorts displayed the weakest neutralization against BA.4/5, with NT_50_ values being reduced to 4.6, accompanied by lower numbers of positive neutralizers. Additionally, salivary neutralization against wild-type virus was strongest in vaccinated and BA.2 convalescent subjects, but this elevated neutralization efficiency was lost when challenged with BA.4/5. Our data support the contention that current coronavirus disease 2019 (COVID-19) vaccines efficiently induce humoral immunity. However, antiviral effectiveness in serum and saliva is greatly reduced against novel variants of concern. These results suggest an adjustment of current vaccine strategies to an adapted or alternative vaccine delivery, such as mucosal booster vaccinations, which might establish enhanced or even sterilizing immunity against novel SARS-CoV-2 variants.

**IMPORTANCE** Rising incidences of breakthrough infections caused by SARS-CoV-2 Omicron BA.4/5 have been observed. Although various studies were conducted investigating neutralizing antibodies in sera, mucosal immunity was barely evaluated. Here, we investigated mucosal immunity, since the presence of neutralizing antibodies at mucosal entry sites plays a fundamental role in disease limitation. We found strong induction of serum IgG/IgA, salivary IgA, and neutralization against SARS-CoV-2 wild-type virus in vaccinated/convalescent subjects but detected 10-fold reduced (albeit positive) serum neutralization against BA.4/5. Interestingly, vaccinated and BA.2 convalescent patients demonstrated the greatest serum neutralization against BA.4/5, but this advantageous neutralizing effect was not observed in the saliva. Our data support the contention that current COVID-19 vaccines are very efficient against severe/critical disease progression. Moreover, these results suggest an adjustment of the current vaccine strategy to adapted and alternative vaccine delivery, such as mucosal booster vaccinations, to establish robust sterilizing immunity against novel SARS-CoV-2 variants.

## INTRODUCTION

Since the onset of the severe acute respiratory syndrome coronavirus 2 (SARS-CoV-2) pandemic, the global population has been exposed to several variants differing in transmission, virulence, and immune evasion (1). By the end of 2021, the first Omicron cases were identified in numerous European countries (2, 3). Initially, the BA.1 sublineage of the Omicron variant (BA.1) represented the dominant virus, which was replaced in central Europe by the Omicron BA.2 variant (BA.2) in early 2022. As of June in the same year, the novel emergent Omicron BA.4 (BA.4) and BA.5 (BA.5) sublineages were the leading causes of rising incidences and breakthrough infections in most of Europe, as well as in some parts of Africa and Southeast Asia (4, 5). The key difference between Omicron and previous variants of concern (VOCs), such as the Delta variant, remains the large accumulation of mutations in the receptor binding domain (RBD) of the viral spike (S) protein, which have been associated with enhanced immune escape and transmissibility (6, 7). While 4 mutations in the spike RBD were found in the Delta variant, 15 mutations were identified in Omicron BA.1 (8). The BA.4 and BA.5 variants share the exact same S protein sequence and differ only outside the S coding region, harboring mutations mainly found in BA.2 (8). In contrast to BA.2, the BA.4 and BA.5 sublineages carry the additional spike RBD mutations 69–70del, L452R, and F486V, as well as the R493Q substitution (8, 9). Overall, Omicron variants demonstrate lower hospitalization rates and milder disease progression, compared to earlier VOCs (10). However, with 1.9 million new cases and more than 5,500 deaths per week as of October 2022, the situation in Europe remains serious and difficult to assess (11). Recovery of fully vaccinated individuals, who received two doses of either vector- or mRNA-based vaccines, from early SARS-CoV-2 variants (i.e., Alpha, Beta, or Delta) was associated with enhanced protection against reinfection. This aspect was confirmed to be drastically lower for Omicron BA.1 and BA.2 due to the enhanced immune evasion (12). In a recent study, twice-vaccinated and convalescent individuals demonstrated the strongest immunity against BA.2 and BA.4/5 variants, compared to recipients of heterologous or homologous booster vaccines (13). As of October 2022, 67% of Europeans were fully vaccinated and only 45% had received a booster dose despite the promising opportunity to increase protection against VOCs (14). With the current increases in SARS-CoV-2 BA.4/5 incidences, evaluation of immunity elicited by BA.1 or BA.2 is of great interest to predict the potential infection rate. Moreover, understanding the level of protection within the population induced by vaccination and/or convalescence against current VOCs allows an assessment of the reintroduction of nonpharmaceutical coronavirus disease 2019 (COVID-19) interventions such as region-wide lockdowns or mass testing. In this work, we compared the humoral immune responses of 92 individuals, including boosted and BA.1-recovered (Vac/BA.1) (see Table S1 in the supplemental material) or BA.2-recovered (Vac/BA.2) subjects (see Table S2), boosted but nonconvalescent (Vac) subjects (see Table S3), and unvaccinated BA.1 convalescents (BA.1-conv) (see Table S4). We examined RBD-specific IgG levels in serum samples and S1-specific IgA levels in serum and saliva samples. In addition, we determined half-maximal neutralizing capacity (50% neutralization titer [NT_50_]) against replication-competent SARS-CoV-2 wild-type (WT) and Omicron BA.4/5 viruses, using both serum and saliva samples to investigate humoral protection against BA.4/5 in differently immunized (vaccinated and/or infected) individuals.

## RESULTS

### Determining IgG and IgA titers in vaccinated and/or convalescent individuals.

**FIG 1:**
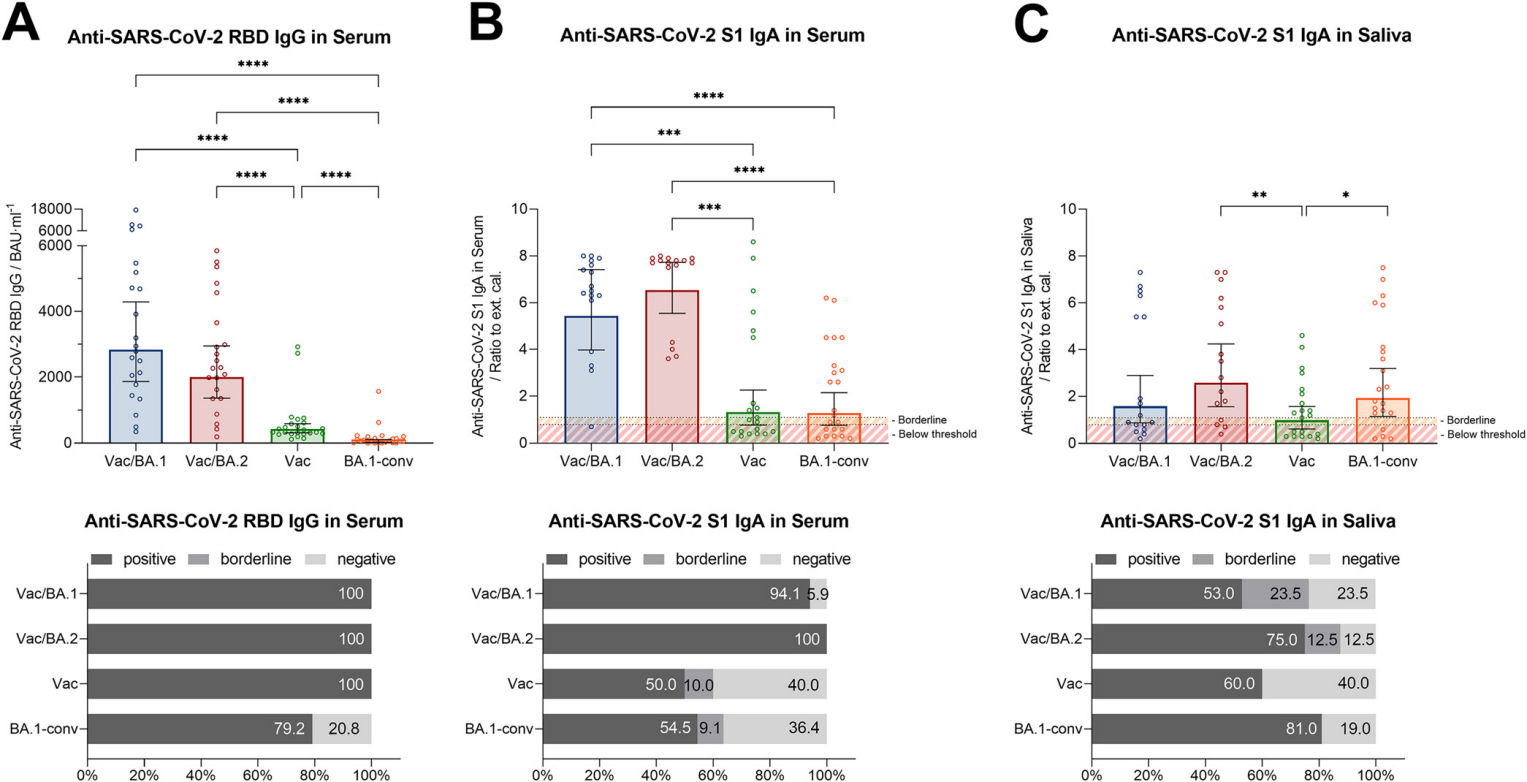
Analyses of SARS-CoV-2-specific antibodies in sera and saliva from vaccinated and/or convalescent individuals. (Upper) Serum was tested for SARS-CoV-2 RBD-specific IgG (A) and S1-specific IgA (B). Additionally, the presence of S1-specific IgA was determined in saliva (C). IgG and IgA titers from three times vaccinated and BA.1 (Vac/BA.1) (blue) or BA.2 (Vac/BA.2) (red) convalescent individuals, as well as three times vaccinated but nonconvalescent individuals (Vac) (orange) and nonvaccinated but BA.1 convalescent individuals (BA.1-conv) (green), are shown. Red lines indicate positive titer thresholds, and yellow lines indicate borderline levels. (Lower) Percentages of individuals with positive, borderline, and negative IgG or IgA titers are shown. Thresholds for IgG and IgA were set according the manufacturer’s instructions (WT IgG against RBD, positive is >7.1 BAU/mL; WT IgA against S1, positive ratio is >1.1 and borderline ratio is 0.9 to 1.1). Data are shown as geometric means ± 95% confidence intervals (CIs), and statistical significances were determined via the Mann-Whitney test. *, *P* < 0.05; **, *P* < 0.01; ***, *P* < 0.001; ****, *P* < 0.0001.

Positive titers of anti-SARS-CoV-2 RBD-specific IgG were detected in all tested groups (100%) except the BA.1-conv cohort, which showed a 21% decrease (Fig. 1A and Table 1). The highest IgG levels were measured in the Vac/BA.1 group (2,830.0 binding antibody units [BAU]/mL), followed by Vac/BA.2 (2,002.0 BAU/mL). In comparison, the mean IgG level of vaccinated but uninfected individuals demonstrated a 9-fold reduction (430.9 BAU/mL) (Fig. 1A and Table 1). The lowest IgG levels were observed in the BA.1-conv group, with a geometric mean of 48.1 BAU/mL (Fig. 1A and Table 1). Due to the possible decrease in sensitivity for Omicron-specific antibodies, especially in the BA.1-conv group, we performed an additional SARS-CoV-2 Omicron S1-specific IgG enzyme-linked immunosorbent assay (ELISA) using serum samples. We determined overall lower levels of Omicron-specific IgG titers, compared to those for the WT virus, but similar trends for the two assays within the tested cohorts (Fig. 1A; also see Fig. S3 in the supplemental material). Determination of SARS-CoV-2 S1-specific serum IgA values (Fig. 1B and Table 1) revealed the highest serum IgA levels for subjects of the Vac/BA.2 and Vac/BA.1 groups, while significantly lower IgA titers were found for the Vac and BA.1-conv cohorts (Fig. 1B, upper, and Table 1). In addition, these groups also demonstrated the smallest proportions of serum IgA-positive subjects (Fig. 1B, lower). Regarding S1-specific salivary IgA, positive titers were generally lower than those for serum but, here also, significantly higher IgA levels were detected in the Vac/BA.2 group, with a geometric mean ratio of 2.6 (*P* = 0.0377) (Fig. 1C, upper, and Table 1). Interestingly, 3×Vac/BA.1 and BA.1-conv groups reached similar salivary IgA levels, with the BA.1-conv group demonstrating slightly higher values and percentages of IgA-positive individuals (Fig. 1C and Table 1). The lowest salivary IgA responses were detected in Vac subjects (Fig. 1C and Table 1). Independent of cohort, very strong correlations were identified for serum IgG versus serum IgA levels, with moderate correlations for serum IgA and salivary IgA levels.

**TABLE 1:** IgG (against SARS-CoV-2 WT RBD) and IgA (against SARS-CoV-2 S1) titers in serum and saliva samples

Ig isotype (target region) and group	IgG or IgA titer (geometric mean [95% CI])^*a*^
Serum IgG (against WT RBD) (BAU/mL)	
Vac/BA.1	2,830.0 (1,866.0–4,291.0)
Vac/BA.2	2,002.0 (1,361.0–2,645.0)
Vac	430.9 (313.5–592.1)
BA.1-conv	48.1 (21.3–145.4)
Serum IgA (against WT S1)	
Vac/BA.1	5.4 (4.0–7.4)
Vac/BA.2	6.5 (5.5–7.7)
Vac	1.3 (0.8–2.3)
BA.1-conv	1.3 (0.8–2.1)
Saliva IgA (against WT S1)	
Vac/BA.1	1.6 (0.9–2.9)
Vac/BA.2	2.6 (1.6–4.2)
Vac	1.0 (0.6–1.6)
BA.1-conv	1.9 (1.2–3.2)

a Antibody titers are shown as geometric means with 95% confidence intervals (CIs) for serum IgG against SARS-CoV-2 RBD (BAU per milliliter) or IgA against S1 (external control/external calibrator) in serum and saliva samples.

### Determining neutralizing capacities of serum and saliva samples in vaccinated and/or convalescent individuals.

**FIG 2:**
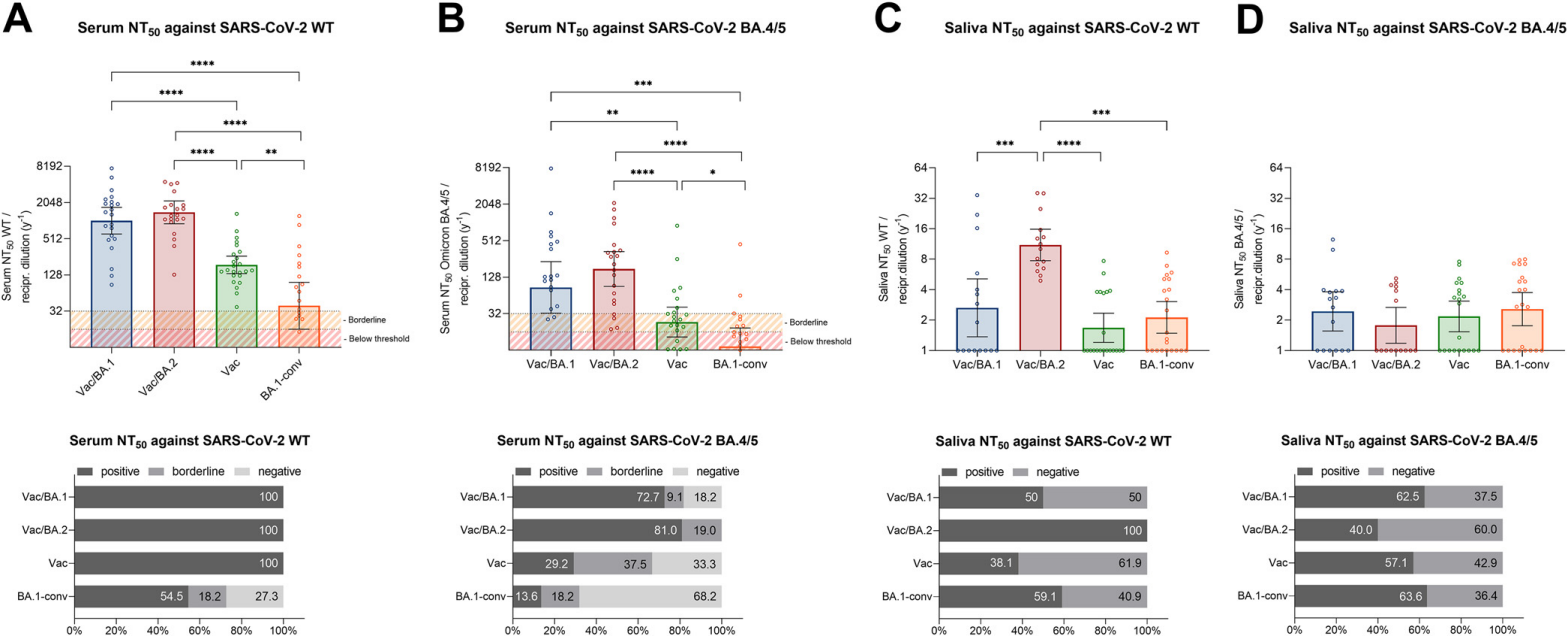
Analyses of neutralization capacity using sera and saliva from vaccinated and/or convalescent individuals. (Upper) Graphs illustrate serum neutralization against the WT virus (A) and the BA.4/5 variant (B) and salivary neutralization against the WT virus (C) and the BA.4/5 variant (D). Data for three times vaccinated and BA.1 (Vac/BA.1) (blue) or BA.2 (Vac/BA.2) (red) convalescent individuals, three times vaccinated but nonconvalescent individuals (Vac) (orange), and nonvaccinated but BA.1 convalescent individuals (BA.1-conv) (green) are shown. Red lines indicate the defined neutralization threshold, and yellow lines indicate borderline levels. (Lower) Percentages of individuals in each group with positive, borderline, and negative neutralization are presented. Thresholds for sera were defined as positive for NT_50_ values of >32 and as borderline for NT_50_ values between 16 and 32. For saliva, NT_50_ thresholds were set as positive for NT_50_ values of >1. Data are shown as geometric means ± 95% CIs, and statistical significances were determined via the Mann-Whitney test. *, *P* < 0.05; **, *P* < 0.01; ***, *P* < 0.001; ****, *P* < 0.0001.

No correlative relationship was found between serum IgG and salivary IgA levels (see Fig. S1). To determine the neutralizing capacities of antibodies in serum and saliva samples, we conducted NT_50_ analyses with replication-competent SARS-CoV-2 WT and Omicron BA.4/5 viruses. The highest serum neutralization titers were found in the Vac/BA.2 and Vac/BA.1 cohorts, followed by the boosted group, with NT_50_ values of 1,409.0, 1,023.0, and 187.8, respectively (Fig. 2A and Table 2). All subjects in the three convalescent groups (Vac/BA.1, Vac/BA.2, and BA.1-conv) demonstrated high neutralization titers (Fig. 2A, lower). However, reduced neutralization was shown by BA.1 convalescent patients, given that geometric mean NT_50_ values reached 39.3, with 54.5% positive neutralization (Fig. 2A). A pattern of serum neutralization similar to that against the WT virus was detected for the BA.4/5 strain. Again, the Vac/BA.2 and Vac/BA.1 cohorts demonstrated the strongest neutralization, with NT_50_ values of 174.2 and 86.6, respectively (Fig. 2B and Table 2); however, neutralization was reduced up to 11-fold, in contrast to that against the WT virus (Fig. 2A and B). Although effective neutralization could be detected against the WT virus in the BA.1 convalescent cohort, only 13.6% of individuals demonstrated positive neutralizing activity against BA.4/5 (Fig. 2B and Table 2). Both the BA.1-conv and Vac cohorts displayed the weakest neutralization against the BA.4/5 strain, with NT_50_ values between 4.6 and 41.0, which was accompanied by low percentages of positive neutralizers in these groups (Table 2). In a next step, we used saliva samples from all four cohorts and determined neutralizing capacities against the WT virus and BA4/5. We found that the highest salivary NT_50_ values were measured in the Vac/BA.2 group, while all other cohorts demonstrated significantly lower neutralization titers (Fig. 2C and Table 2). Similar NT_50_ values were shown for the Vac/BA.1 and BA.1-conv cohorts, while the boosted cohort had the lowest titers (Fig. 2C and Table 2). Nevertheless, the elevated neutralization against the WT virus observed in Vac/BA.2 individuals diminished against BA.4/5 and decreased to the same level as in the other cohorts (Fig. 2D and Table 2). Thus, no significant differences could be found between the cohorts (Fig. 2D). To test whether heterologous or homologous booster regimens could affect our observations, we compared individuals within each group who had received either two doses of the vector-based vaccine followed by an mRNA-based booster or two doses of an mRNA-based vaccine followed by an mRNA-based booster. We found no significant differences in antibody titers or NT_50_ values between these groups (data not shown). Overall, rates of positive salivary neutralization against the WT virus and BA.4/5 ranged from 38.1% to 100% and from 40.0 to 63.6%, respectively (Fig. 2D, lower). Spearman correlation of all tested groups demonstrated a positive and significant correlation between serum antibodies and serum neutralization (see Fig. S4). Furthermore, positive correlation for salivary IgA and salivary neutralization was found only against the WT virus and not against BA.4/5 (see Fig. S4).

**TABLE 2:** NT_50_ against SARS-CoV-2 WT virus or BA.4/5 in serum and saliva samples

Specimen type and virus variant	Group	NT_50_ (geometric mean [95% CI])^*a*^
Serum		
WT virus	Vac/BA.1	1,023.0 (612.1–1,708.0)
	Vac/BA.2	1,409.0 (909.3–2,183.0)
	Vac	187.8 (134.2–262.9)
	BA.1-conv	39.3 (16.0–96.4)
Omicron BA.4/5	Vac/BA.1	86.6 (32.6–230.1)
	Vac/BA.2	174.2 (90.1–336.8)
	Vac	23.2 (13.1–41.0)
	BA.1-conv	9.2 (4.6–18.5)
Saliva		
WT virus	Vac/BA.1	2.6 (1.4–5.1)
	Vac/BA.2	11.6 (7.7–15.8)
	Vac	2.1 (1.5–3.0)
	BA.1-conv	1.6 (1.2–2.3)
Omicron BA.4/5	Vac/BA.1	2.4 (1.6–3.8)
	Vac/BA.2	1.8 (1.2–2.7)
	Vac	2.2 (1.5–3.1)
	BA.1-conv	2.6 (1.8–3.7)

a NT_50_ values (reciprocal dilutions) are presented as geometric means with 95% CIs for the Vac/BA.1, Vac/BA.2, Vac, and BA.1-conv groups.

## DISCUSSION

Although several studies compared the spike IgG and neutralizing antibody responses to SARS-CoV-2 variants, only a few investigated spike IgA levels in serum and mucosal sites, and even fewer studied neutralizing antibodies in the mucosal secretions. Here, we determined RBD-specific IgG and S1-specific IgA levels in serum, as well as SARS-CoV-2 S1-specific IgA levels in saliva, and also evaluated salivary and serum neutralization capacities against the WT virus and BA.4/5 using replication competent viruses (15). In particular, we directly compared humoral immune responses of boosted and BA.1 or BA.2 convalescent individuals, boosted but nonconvalescent individuals, and unvaccinated and BA.1 convalescent individuals.

We and others previously demonstrated that SARS-CoV-2-specific IgA can be detected in serum after intramuscular immunization of COVID-19 vaccines (16). Here, we detected similar patterns for SARS-CoV-2 RBD-specific IgG and S1-specific serum IgA, since the Vac/BA.2 and Vac/BA.1 groups showed the highest serum IgA levels. Therefore, our data indicate that infection of vaccinated individuals resulted in stronger serum IgG and IgA titers, compared to only vaccinated or convalescent subjects. The decreased titer in the Vac group is explained by there being no direct contact of virus with the respiratory surface, resulting in weaker mucosal immunity than after infection (17, 18).

For analyses of mucosal IgA titers and immunity, we collected and used saliva samples according to a well-established and standardized in-house protocol (15). The use of saliva samples offers several advantages and, since saliva covers the pharynx, which is part of the SARS-CoV-2 infection pathway, it is suitable for the study of mucosal immunity, as shown previously (19). Upon testing SARS-CoV-2 S1-specific IgA levels in saliva samples from our study participants, we found positive salivary IgA titers in all three convalescent groups but not in the Vac group. A few studies also examined salivary IgA titers after COVID-19 vaccination or recovery (15), but increasing mucosal IgA through alternative vaccination strategies might be a key for protective immunity against SARS-CoV-2. In fact, recent data in mice revealed strong induction of secretory IgA in the mucosa following an intranasal boost after initial priming. In addition, that study showed that tissue-resident memory T cells that could completely prevent infection with different SARS-CoV-2 variants after exposure were generated (20, 21).

Furthermore, we determined the neutralizing capacity (NT_50_) against replication-competent SARS-CoV-2 viruses using both serum and saliva samples, to investigate protection against WT and BA.4/5 viruses in differently immunized individuals. The highest serum neutralization titers against the WT virus and to a lesser extend also BA.4/5 were found in the Vac/BA.2 and Vac/BA.1 cohorts, while reduced neutralization was shown by Vac and BA.1-conv subjects. Spearman analyses confirmed a very strong or strong and significant correlation between serum IgG and serum NT_50_ values against the WT virus and BA.4/5, respectively. These results are consistent with titers of similar cohorts and indicate that vaccination in combination with infection provides moderate but detectable humoral immune response against the current BA.4/5 virus (22). In contrast, neutralization experiments using saliva revealed that Vac/BA.2 individuals showed significantly elevated neutralizing capacity against the WT virus, which is in accordance with the significantly higher titers of saliva IgA that were induced by this combination. The elevated neutralizing effect was not observed against BA.4/5, indicating that BA.2 infection improves salivary IgA levels but does not improve protection against BA.4/5. Spearman calculations confirmed that high salivary IgA titers also improved neutralization against the WT virus; however, such conclusions could not be drawn for BA.4/5. These findings are striking, because they show that previous infection with BA.1 or BA.2 after vaccination results in strong serum antibody induction that could limit disease progression for BA.4/5. However, this natural immunization has no beneficial effect on the neutralizing capacity in saliva against this virus variant, although it is highly effective against the WT virus. Elevated serum antibody titers represent a possible explanation for the current low rate of hospitalization and milder disease progression despite increasing incidences (23, 24).

The detected amount of neutralizing antibodies in saliva is probably not sufficient to prevent infection with BA.4/5, but the systemic humoral immune response remains effective in preventing severe or critical COVID-19. Overall, our data emphasize the proposed future vaccine booster strategies from intramuscular to an oral or intranasal approach, which could provide more potent and protective immunity, as demonstrated in animal experiments (20, 21). Furthermore, the use of adapted vaccines might be of advantage, since our data indicated that cohorts were protected against reinfection with the WT virus but not against infection with novel SARS-CoV-2 variants such as BA.4/5 (25). Potential limitations of our study could include the variation of sampling days after the last immunization for the vaccinated but uninfected group. Moreover, the ELISA kit used to determine IgG/IgA titers was designed to measure WT virus-specific antibodies, which might diminish the results, especially for the BA.1-conv group. Although we expanded antibody testing to include SARS-CoV-2 Omicron S1-specific antibodies in serum, at the time there was no saliva-validated IgA ELISA for SARS-CoV-2 RBD- or variant-specific Ig detection available.

## MATERIALS AND METHODS

### Ethics statement.

Written informed consent was obtained from all donors for leftover nasopharyngeal/oropharyngeal specimens, serum samples, and saliva samples by the participating clinics. The Ethics Committee of the Medical University of Innsbruck approved the use of anonymized leftover specimens from COVID-19 patients and vaccinees (ethics approval number ECS1166/2020) and healthy donors (ethics approval number ECS1166/2018) for scientific purposes.

### Human samples.

In this study, serum and saliva samples from 92 individuals were collected and divided into four groups, as follows: group Vac/BA.1, three times vaccinated and SARS-CoV-2 BA.1 convalescent (*n* = 22) (see Table S1 and Fig. S1 in the supplemental material); group Vac/BA.2, three times vaccinated and SARS-CoV-2 BA.2 convalescent (*n* = 22) (see Table S2 and Fig. S1); group Vac, three times vaccinated and nonconvalescent (*n* = 24) (see Table S3 and Fig. S1); group BA.1-conv, unvaccinated but SARS-CoV-2 BA.1 convalescent (*n* = 24) (see Table S4 and Fig. S1). The geometric mean age of all participants was 36.7 years, and the proportions of male and female subjects were 58.7% and 41.3%, respectively. All vaccinated individuals were vaccinated with two doses of ChAdOx1 (AstraZeneca), mRNA-1273 (Moderna), or BNT162b2 (BioNTech/Pfizer), followed by a heterologous or homologous booster of either mRNA-1273 or BNT162b2. All included COVID-19 patients were diagnosed by PCR and showed mild disease severity, which did not require any treatment or hospitalization. Virus variants of convalescent individuals were confirmed via mutant-specific PCR or inferred on the basis of the date of diagnosis (first positive PCR test) and the prevalence of the current virus variant in the specific region, as provided by the GISAID database (covariants.org) (see Fig. S2). The geometric mean sampling day after the last immunization (vaccination or infection) or days between the third vaccination and infection for groups Vac/BA.1 and Vac/BA.2 were 61.5 and 114.6 days, respectively (see Fig. S1A). The geometric means of days between the last vaccination/infection and sampling for groups Vac/BA.1, Vac/BA.2, and Ba.1-conv were 48.2, 67.4, and 60.3 days (see Fig. S1B). For the 3×Vac group, the geometric mean of days between the last vaccination and sampling was 196.0 days (see Fig. S1B).

### Viruses.

SARS-CoV-2 WT virus (Centers for AIDS Research [CFAR]/National Institute for Biological Standards and Control [NIBSC] number 52281) was obtained from a repository (BEI Resources, Manassas, VA, USA) and propagated according to the manufacturer’s instructions. Clinical specimens for SARS-CoV-2 Omicron (B.1.1.529) BA.4/5 were isolated from COVID-19-positive swabs, and the variant was confirmed via mutant-specific PCR (ethics approval number ECS1166/2020) (see above). Virus was subsequently cultured as described previously (16).

### Determination of antibody titers in serum and saliva samples against S1 and RBD from SARS-CoV-2 spike protein.

Serum samples from vaccinated or COVID-19 convalescent participants were retrieved from blood samples in serum collection tubes by centrifugation at 300 × *g* for 5 min, and serum fractions were collected and stored at −80°C until use. Saliva samples were collected using Salivette saliva collection tubes (Sarstedt, Nümbrecht, Germany). As suggested by the manufacturer, the liquid phase was obtained after centrifugation at 4,000 × *g* for 5 min and was stored at −80°C until use. Serum and saliva samples were analyzed with the SARS-CoV-2 IgG II Quant assay (Abbott, USA). The chemiluminescent microparticle immunoassay (CMIA) SARS-CoV-2 IgG II Quant assay was performed in order to assess anti-SARS-CoV-2 IgG levels against WT RBD. CMIA results were calculated as BAU per milliliter, and the cutoff value for positive results was set at 7.1 BAU/mL according to the manufacturer’s instructions. IgA antibody titers in sera and saliva were analyzed via an ELISA (Euroimmun, Lübeck, Germany) for anti-SARS-CoV-2 IgA against WT S1 protein. Results are shown as a ratio (external control/external calibrator), and the manufacturer defines a ratio of ≥1.1 as positive. Levels of anti-Omicron BA.1 IgG against S1 protein were determined by using the QuantiVac ELISA (Euroimmun), which allows conversion of the relative units (RU) per milliliter to BAU per milliliter. Unit conversion was performed according to the manufacturer’s instructions, resulting in 35.2 BAU/mL as the positive cutoff value and 35.2 to 25.6 BAU/mL as the borderline range.

### Immunofluorescence neutralization assay.

VeroE6-TMPRSS2-ACE2 cells (2 × 10^4^ cells) were seeded in a 96-well plate with culture medium (high-glucose Dulbecco’s modified Eagle‘s medium [DMEM] supplemented with 10% fetal calf serum [FCS], 1% l-glutamine, and 1% penicillin-streptomycin; all reagents were obtained from Sigma-Aldrich, St. Louis, MO, USA), and cultures were incubated overnight at 37°C in 5% CO_2_. After 2 days in culture, heat-inactivated serum and saliva samples were serially diluted from 1:8 to 1:8,192 or from 1:4 to 1:512, respectively. Dilutions were incubated with SARS-CoV-2 WT virus or BA.4/5 variant (2.5 × 10^2^ PFU/mL) for 1 h at 37°C, and then dilutions were inoculated into VeroE6-TMPRSS2-ACE2 cells for 1 h at 37°C in 5% CO_2_. After incubation, the inoculum was aspirated, and the cells were washed with Dulbecco’s phosphate-buffered saline (D-PBS) and incubated at 37°C in 5% CO_2_ in DMEM supplemented with 1.5% FCS, 1% l-glutamine, and 1% penicillin-streptomycin. After 16 h, the medium was removed and the cells were fixed in 4% formalin (Sigma-Aldrich) for 30 min at room temperature. After fixation, the cells were permeabilized (intracellular staining permeabilization wash buffer; BioLegend, San Diego, CA, USA) according to the manufacturer’s instructions. The same buffer was used for immunofluorescence staining with primary (monoclonal rabbit anti-SARS-CoV-2 nucleocapsid antibody; Sinobiological, China) and secondary (goat anti-rabbit IgG conjugated to Alexa Fluor 488; Invitrogen, USA) antibodies. Cells were washed twice in D-PBS before imaging with the Operetta CLS microscope (Perkin Elmer, Waltham, MA, USA). Image analysis and quantification of infected areas were performed using Harmony software v4.8 (Perkin Elmer). NT_50_ values from neutralization curves were calculated using four-parameter nonlinear regression in GraphPad Prism v9. Values of 1:32 for serum and 1:1 for saliva were defined as cutoff values for positive neutralization.

### Statistical analysis.

Statistical determination of cutoff values for antibody titers (serum IgG, serum IgA, and salivary IgA) were provided by the manufacturer and applied as suggested. Statistical analysis was performed using GraphPad Prism v9. Significances of antibody titers and NT_50_ values were determined via the Kruskal-Wallis test with Dunn’s multiple correction. Correlations were computed using a two-tailed nonparametric Spearman test.

## References

[1] Lazarevic I, Pravica V, Miljanovic D, Cupic M. 2021. Immune evasion of SARS-CoV-2 emerging variants: what have we learnt so far? Viruses 13:1192. doi:10.3390/v13071192. 34206453 PMC8310325

[2] Nyberg T, Ferguson NM, Nash SG, Webster HH, Flaxman S, Andrews N, Hinsley W, Bernal JL, Kall M, Bhatt S, Blomquist P, Zaidi A, Volz E, Aziz NA, Harman K, Funk S, Abbott S, Hope R, Charlett A, Chand M, Ghani AC, Seaman SR, Dabrera G, De Angelis D, Presanis AM, Thelwall S, COVID-19 Genomics UK (COG-UK) Consortium. 2022. Comparative analysis of the risks of hospitalisation and death associated with SARS-CoV-2 omicron (B.1.1.529) and delta (B.1.617.2) variants in England: a cohort study. Lancet 399:1303–1312. doi:10.1016/S0140-6736(22)00462-7. 35305296 PMC8926413

[3] Brandal LT, MacDonald E, Veneti L, Ravlo T, Lange H, Naseer U, Feruglio S, Bragstad K, Hungnes O, Ødeskaug LE, Hagen F, Hanch-Hansen KE, Lind A, Watle SV, Taxt AM, Johansen M, Vold L, Aavitsland P, Nygård K, Madslien EH. 2021. Outbreak caused by the SARS-CoV-2 Omicron variant in Norway, November to December 2021. Euro Surveill 26:2101147. doi:10.2807/1560-7917.ES.2021.26.50.2101147. 34915975 PMC8728491

[4] Hodcroft E. 2022. Overview of variants/mutations. https://covariants.org/per-variant?variant=22A+%28Omicron%29&variant=22B+%28Omicron%29. Accessed 13 July 2022.

[5] Takashita E, Yamayoshi S, Simon V, van Bakel H, Sordillo EM, Pekosz A, Fukushi S, Suzuki T, Maeda K, Halfmann P, Sakai-Tagawa Y, Ito M, Watanabe S, Imai M, Hasegawa H, Kawaoka Y. 2022. Efficacy of antibodies and antiviral drugs against Omicron BA.2.12.1, BA.4, and BA.5 subvariants. N Engl J Med 387:468–470. doi:10.1056/NEJMc2207519. 35857646 PMC9342381

[6] Torjesen I. 2021. Covid-19: Omicron may be more transmissible than other variants and partly resistant to existing vaccines, scientists fear. BMJ 375:n2943. doi:10.1136/bmj.n2943. 34845008

[7] Callaway E. 2021. Heavily mutated Omicron variant puts scientists on alert. Nature 600:21. doi:10.1038/d41586-021-03552-w. 34824381

[8] Tegally H, Moir M, Everatt J, Giovanetti M, Scheepers C, Wilkinson E, Subramoney K, Makatini Z, Moyo S, Amoako DG, Baxter C, Althaus CL, Anyaneji UJ, Kekana D, Viana R, Giandhari J, Lessells RJ, Maponga T, Maruapula D, Choga W, Matshaba M, Mbulawa MB, Msomi N, NGS-SA Consortium, Naidoo Y, Pillay S, Sanko TJ, San JE, Scott L, Singh L, Magini NA, Smith-Lawrence P, Stevens W, Dor G, Tshiabuila D, Wolter N, Preiser W, Treurnicht FK, Venter M, Chiloane G, McIntyre C, O'Toole A, Ruis C, Peacock TP, Roemer C, Kosakovsky Pond SL, Williamson C, Pybus OG, Bhiman JN, Glass A, Martin DP, Jackson B, Rambaut A, Laguda-Akingba O, Gaseitsiwe S, von Gottberg A, de Oliveira T. 2022. Emergence of SARS-CoV-2 Omicron lineages BA.4 and BA.5 in South Africa. Nat Med 28:1785–1790. doi:10.1038/s41591-022-01911-2. 35760080 PMC9499863

[9] Tuekprakhon A, Nutalai R, Dijokaite-Guraliuc A, Zhou D, Ginn HM, Selvaraj M, Liu C, Mentzer AJ, Supasa P, Duyvesteyn HME, Das R, Skelly D, Ritter TG, Amini A, Bibi S, Adele S, Johnson SA, Constantinides B, Webster H, Temperton N, Klenerman P, Barnes E, Dunachie SJ, Crook D, Pollard AJ, Lambe T, Goulder P, Paterson NG, Williams MA, Hall DR, OPTIC Consortium, ISARIC4C Consortium, Fry EE, Huo J, Mongkolsapaya J, Ren J, Stuart DI, Screaton GR. 2022. Antibody escape of SARS-CoV-2 Omicron BA.4 and BA.5 from vaccine and BA.1 serum. Cell 185:2422–2433.e13. doi:10.1016/j.cell.2022.06.005. 35772405 PMC9181312

[10] Nealon J, Cowling BJ. 2022. Omicron severity: milder but not mild. Lancet 399:412–413. doi:10.1016/S0140-6736(22)00056-3. 35065007 PMC8769661

[11] World Health Organization. 2022. WHO (COVID-19) dashboard. https://covid19.who.int.

[12] Altarawneh HN, Chemaitelly H, Hasan MR, Ayoub HH, Qassim S, AlMukdad S, Coyle P, Yassine HM, Al-Khatib HA, Benslimane FM, Al-Kanaani Z, Al-Kuwari E, Jeremijenko A, Kaleeckal AH, Latif AN, Shaik RM, Abdul-Rahim HF, Nasrallah GK, Al-Kuwari MG, Butt AA, Al-Romaihi HE, Al-Thani MH, Al-Khal A, Bertollini R, Tang P, Abu-Raddad LJ. 2022. Protection against the Omicron variant from previous SARS-CoV-2 infection. N Engl J Med 386:1288–1290. doi:10.1056/NEJMc2200133. 35139269 PMC8849180

[13] Jäger M, Diem G, Sahanic S, Fux V, Griesmacher A, Lass-Flörl C, Wilflingseder D, Tancevski I, Posch W. 2023. Immunity of heterologous and homologous boosted or convalescent individuals against Omicron BA.1, BA.2 and BA.4/5 variants. J Infect Dis doi:10.1093/infdis/jiad057. PMC10345468 36869832

[14] Our World in Data. 2022. COVID-19 vaccine doses, people with at least one dose, people with a full initial protocol, and boosters per 100 people, Aug 19, 2022. https://ourworldindata.org/explorers/coronavirus-data-explorer?time=2022-08-19&uniformYAxis=0&Metric=Vaccine+doses%2C+people+vaccinated%2C+and+booster+doses&Interval=7-day+rolling+average&Relative+to+Population=true&Color+by+test+positivity=false&country=Africa~Europe~Asia~North+America~Oceania~South+America~OWID_WRL. Accessed 19 August 2022.

[15] Diem G, Lafon E, Bauer A, Lass-Flörl C, Reindl M, Wilflingseder D, Posch W. 2022. Salivary IgAs and their role in mucosal neutralization of SARS-CoV-2 variants of concern. J Clin Microbiol 60:e01065-22. doi:10.1128/jcm.01065-22. 36036600 PMC9491179

[16] Lafon E, Jäger M, Bauer A, Reindl M, Bellmann-Weiler R, Wilflingseder D, Lass-Flörl C, Posch W. 2022. Comparative analyses of IgG/IgA neutralizing effects induced by three COVID-19 vaccines against variants of concern. J Allergy Clin Immunol 149:1242–1252.e12. doi:10.1016/j.jaci.2022.01.013. 35093484 PMC8799473

[17] Jeyanathan M, Afkhami S, Smaill F, Miller MS, Lichty BD, Xing Z. 2020. Immunological considerations for COVID-19 vaccine strategies. Nat Rev Immunol 20:615–632. doi:10.1038/s41577-020-00434-6. 32887954 PMC7472682

[18] Azzi L, Dalla Gasperina D, Veronesi G, Shallak M, Ietto G, Iovino D, Baj A, Gianfagna F, Maurino V, Focosi D, Maggi F, Ferrario MM, Dentali F, Carcano G, Tagliabue A, Maffioli LS, Accolla RS, Forlani G. 2022. Mucosal immune response in BNT162b2 COVID-19 vaccine recipients. EBioMedicine 75:103788. doi:10.1016/j.ebiom.2021.103788. 34954658 PMC8718969

[19] Guerrieri M, Francavilla B, Fiorelli D, Nuccetelli M, Passali F, Coppeta L, Somma G, Bernardini S, Magrini A, Di Girolamo S. 2021. Nasal and salivary mucosal humoral immune response elicited by mRNA BNT162b2 COVID-19 vaccine compared to SARS-CoV-2 natural infection. Vaccines (Basel) 9:1499. doi:10.3390/vaccines9121499. 34960244 PMC8708818

[20] Lapuente D, Fuchs J, Willar J, Vieira Antão A, Eberlein V, Uhlig N, Issmail L, Schmidt A, Oltmanns F, Peter AS, Mueller-Schmucker S, Irrgang P, Fraedrich K, Cara A, Hoffmann M, Pöhlmann S, Ensser A, Pertl C, Willert T, Thirion C, Grunwald T, Überla K, Tenbusch M. 2021. Protective mucosal immunity against SARS-CoV-2 after heterologous systemic prime-mucosal boost immunization. Nat Commun 12:6871. doi:10.1038/s41467-021-27063-4. 34836955 PMC8626513

[21] An X, Martinez-Paniagua M, Rezvan A, Sefat SR, Fathi M, Singh S, Biswas S, Pourpak M, Yee C, Liu X, Varadarajan N. 2021. Single-dose intranasal vaccination elicits systemic and mucosal immunity against SARS-CoV-2. iScience 24:103037. doi:10.1016/j.isci.2021.103037. 34462731 PMC8388188

[22] Hachmann NP, Miller J, Collier A-RY, Ventura JD, Yu J, Rowe M, Bondzie EA, Powers O, Surve N, Hall K, Barouch DH. 2022. Neutralization escape by SARS-CoV-2 Omicron subvariants BA.2.12.1, BA.4, and BA.5. N Engl J Med 387:86–88. doi:10.1056/NEJMc2206576. 35731894 PMC9258748

[23] Lauring AS, Tenforde MW, Chappell JD, Gaglani M, Ginde AA, McNeal T, Ghamande S, Douin DJ, Talbot HK, Casey JD, Mohr NM, Zepeski A, Shapiro NI, Gibbs KW, Files DC, Hager DN, Shehu A, Prekker ME, Erickson HL, Exline MC, Gong MN, Mohamed A, Johnson NJ, Srinivasan V, Steingrub JS, Peltan ID, Brown SM, Martin ET, Monto AS, Khan A, Hough CL, Busse LW, Ten Lohuis CC, Duggal A, Wilson JG, Gordon AJ, Qadir N, Chang SY, Mallow C, Rivas C, Babcock HM, Kwon JH, Halasa N, Grijalva CG, Rice TW, Stubblefield WB, Baughman A, Womack KN, Rhoads JP, Lindsell CJ, Hart KW, Zhu Y, Adams K, Schrag SJ, Olson SM, Kobayashi M, Verani JR, Patel MM, Self WH, Influenza and Other Viruses in the Acutely Ill (IVY) Network. 2022. Clinical severity of, and effectiveness of mRNA vaccines against, covid-19 from omicron, delta, and alpha SARS-CoV-2 variants in the United States: prospective observational study. BMJ 376:e069761. doi:10.1136/bmj-2021-069761. 35264324 PMC8905308

[24] Sievers C, Zacher B, Ullrich A, Huska M, Fuchs S, Buda S, Haas W, Diercke M, An der Heiden M, Kröger S. 2022. SARS-CoV-2 Omicron variants BA.1 and BA.2 both show similarly reduced disease severity of COVID-19 compared to Delta, Germany, 2021 to 2022. Euro Surveill 27:2200396. doi:10.2807/1560-7917.ES.2022.27.22.2200396. 35656831 PMC9164675

[25] Xu K, Gao P, Liu S, Lu S, Lei W, Zheng T, Liu X, Xie Y, Zhao Z, Guo S, Tang C, Yang Y, Yu W, Wang J, Zhou Y, Huang Q, Liu C, An Y, Zhang R, Han Y, Duan M, Wang S, Yang C, Wu C, Liu X, She G, Liu Y, Zhao X, Xu K, Qi J, Wu G, Peng X, Dai L, Wang P, Gao GF. 2022. Protective prototype-Beta and Delta-Omicron chimeric RBD-dimer vaccines against SARS-CoV-2. Cell 185:2265–2278.e2214. doi:10.1016/j.cell.2022.04.029. 35568034 PMC9042943

