# Supplementary material for: Vaccination and Omicron BA.1/BA.2 Convalescence Enhance Systemic but Not Mucosal Immunity against BA.4/5
Source: Microbiol Spectr. 2023 Apr 26;11(3):e05163-22. doi: 10.1128/spectrum.05163-22 (PMC10269517; doi:10.1128/spectrum.05163-22)
Supplement: Supplemental file 1 — Tables S1 to S4 and Fig. S1 to S4. Download spectrum.05163-22-s0001.pdf, PDF file, 0.6 MB [file spectrum.05163-22-s0001.pdf]

Figure S1: Frequency distribution of days between last vaccination and infection and sampling

A

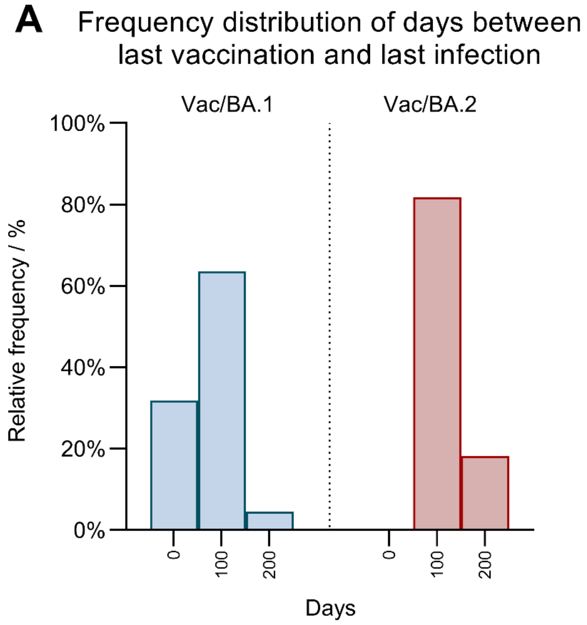

B

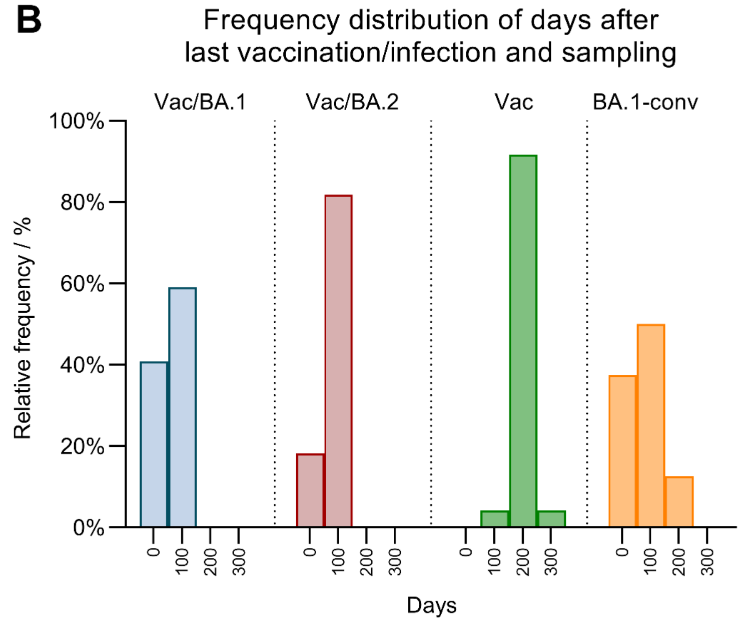

Figure S1: Frequency distribution of days between third vaccination and infection and between last vaccination/infection and sampling.

The histograms shows the frequency distribution of days between third vaccination and infection of groups Vac/BA.1-conv and Vac/BA.2-conv (A) as well as the days between last vaccination or infection and sampling (B) with a bin size of 100 days.

Figure S2: Prevalence of SARS-CoV-2 variants in Austria

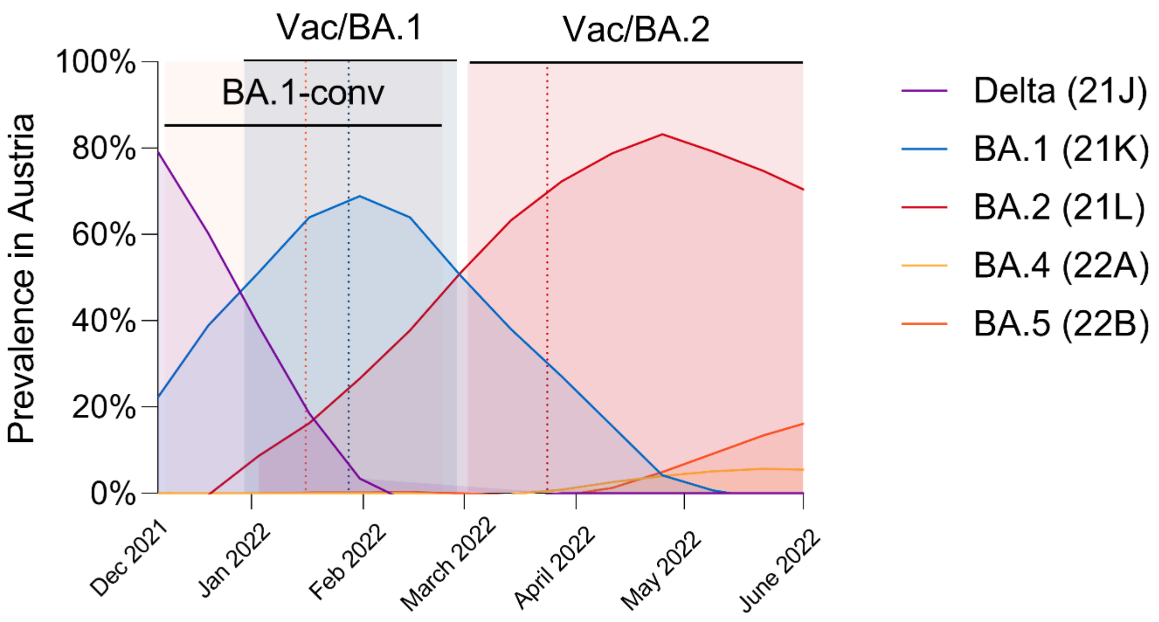

Figure S2: Prevalence of SARS-CoV-2 variants in Austria.

The graph shows the prevalence of SARS-CoV-2 Delta (purple curve), BA.1 (blue curve), BA.2 (red curve), BA.4 (yellow curve) and BA.5 (orange curve) variant in Austria during the period from December 2021 to June 2022. Period of sampling time points of BA.1-conv group is highlighted as yellow area, of Vac/BA.1 group as blue area and of Vac/BA.2 group as red area together with geometric mean sampling time point as dashed lines (yellow, blue and red).

Figure S3: SARS-CoV-2-S1 specific IgG against BA.1 in sera from vaccinated and/or convalescent individuals

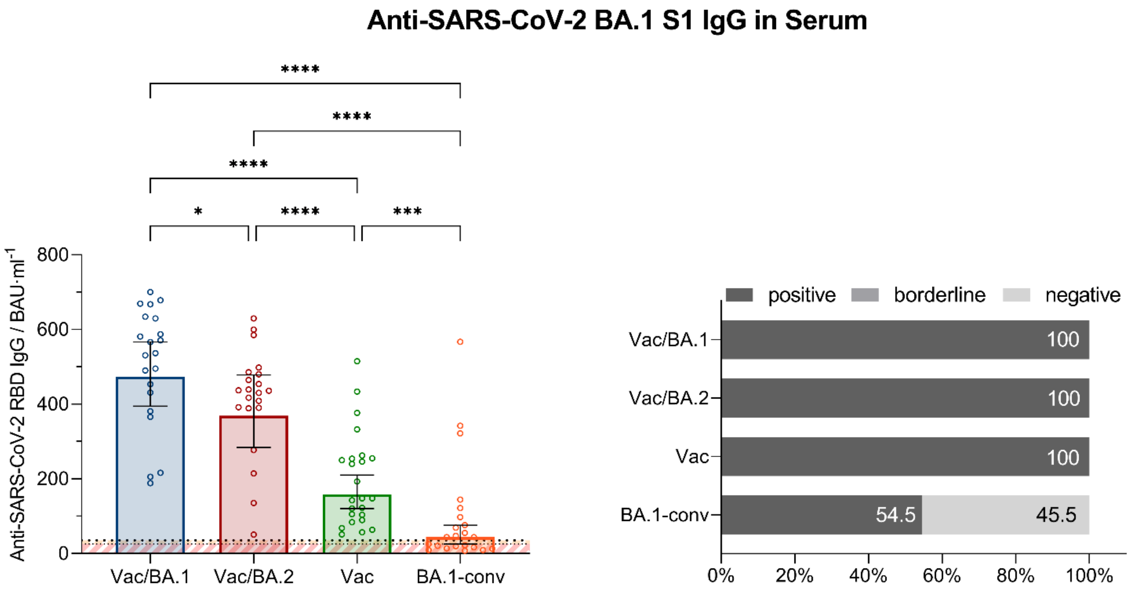

Figure S3: Analyses of SARS-CoV-2-S1 specific IgG against BA.1 in sera from vaccinated and/or convalescent individuals.

(Left panel) Serum was tested for SARS-CoV-2 S1-specific IgG against BA.1. from three times vaccinated and BA.1 (Vac/BA.1, blue) or BA.2 (Vac/BA.2, red) convalescent individuals as well as three times vaccinated but non-convalescent individuals (Vac, orange) and non-vaccinated but BA.1 convalescent individuals (BA.1-conv, green) are shown. Red line indicates positive titer threshold, yellow line indicates borderline level. **(Right panel)** Percentages of individuals with positive, borderline, and negative IgG titers. Thresholds for IgG were set according the manufacturer's instructions (BA.1 IgG against S1: ≥35.2 BAU·ml^{-1} positive, 35.1 - 25.6 BAU·ml^{-1} as borderline). Data is shown as geometric mean ± 95% confidence interval and statistical significances were determined via Mann-Whitney test (*: $p<0.05$, **: $p<0.01$, ***: $p<0.001$, ****: $p<0.0001$).

Figure S4: Spearman r correlation matrix of antibody titers and serum/saliva neutralization

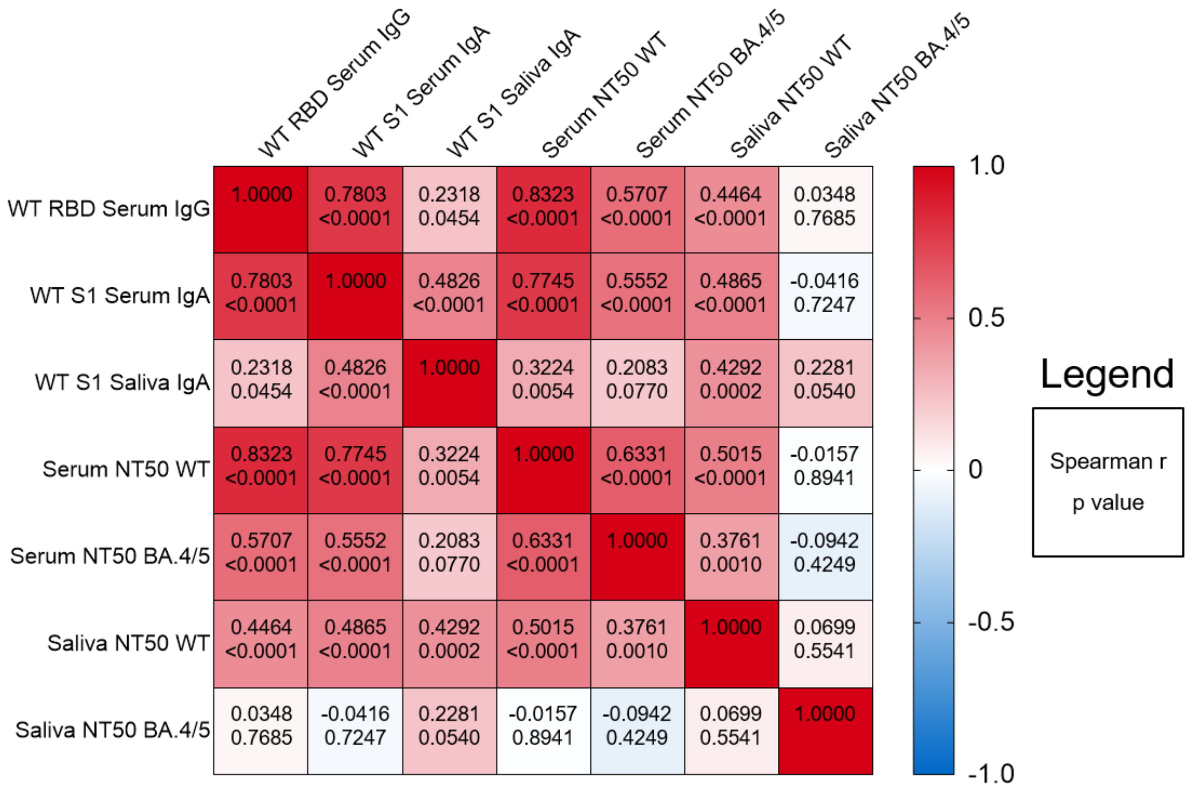

Figure S4: Spearman r correlation matrix of antibody titers and serum/saliva neutralization.

The graphs shows a non-parametric. two-tailed spearman correlation matrix of antibody titers and NT_{50} values of the tested variants. Numbers represent the Spearman r (upper value) and p (lower value).

Table S1: Vaccinated and SARS-CoV-2 BA.1 recovered individuals (Vac/BA.1-conv)

ID	Sex	Age	# of vaccinations (scheme*)	COVID19 recovered	Variant	Date of diagnosis	Days between 3rd vaccination and infection	Days after infection
A1	F	49	3 (AAP)	Yes	BA.1	24.01.2022	69	141
A2	F	29	3 (AAP)	Yes	BA.1	18.01.2022	88	136
A3	F	44	3 (AAM)	Yes	BA.1	24.01.2022	47	134
A4	M	24	3 (AAP)	Yes	BA.1	03.02.2022	58	127
A5	M	42	3 (PPP)	Yes	BA.1	07.02.2022	63	126
A6	M	36	3 (APP)	Yes	BA.1	05.02.2022	70	125
A7	F	32	3 (PPP)	Yes	BA.1	08.02.2022	73	119
A8	M	29	3 (AAP)	Yes	BA.1	13.02.2022	81	114
A9	F	33	3 (AAP)	Yes	BA.1	21.02.2022	83	113
A10	M	60	3 (AAP)	Yes	BA.1	18.02.2022	77	112
A11	F	60	3 (PPP)	Yes	BA.1	21.02.2022	79	109
A12	M	48	3 (AAP)	Yes	BA.1	26.02.2022	100	101
A13	F	35	3 (PPP)	Yes	BA.1	27.02.2022	93	100
A14	F	27	3 (PPP)	Yes	BA.1	30.12.2021	39	28
A15	M	64	3 (PPP)	Yes	BA.1	07.01.2022	140	20
A16	M	59	3 (AAM)	Yes	BA.1	14.02.2022	61	16
A17	M	26	3 (AAP)	Yes	BA.1	10.01.2022	40	15
A18	F	27	3 (PPP)	Yes	BA.1	11.01.2022	29	13
A19	M	35	3 (AAP)	Yes	BA.1	15.01.2022	49	11
A20	M	30	3 (PPP)	Yes	BA.1	16.01.2022	11	9
A21	F	26	3 (PPP)	Yes	BA.1	18.01.2022	151	9
A22	F	29	3 (AAP)	Yes	BA.1	19.01.2022	36	7

n = 22
Age, Geometric mean (CI 95%): 36.5 (5.4)
Sex ratio (M/F): 50% / 50%
Days between 3rd vaccination and infection, Geometric mean (CI 95%): 61.5 (13.1)
Days after infection, Geometric mean (CI 95%): 48.2 (22.6)
*A: ChAdOx1 (AstraZeneca), M: mRNA-1273 (Moderna), P: BNT162 (Biontech Pfizer)

Table S1: Vaccinated and SARS-CoV-2 BA.1 recovered individuals. (Vac/BA.1-conv. n=22).

Sex, age, vaccination/convalescent status, days between 3rd immunization and infection as well as days after infection are presented.

Table S2: Vaccinated and SARS-CoV-2 BA.2 recovered individuals (Vac/BA.2-conv)

ID	Sex	Age	# of vaccinations (scheme*)	COVID19 recovered	Variant	Date of diagnosis	Days between 3rd vaccination and infection	Days after infection
B1	F	34	3 (AAP)	Yes	BA.2	02.03.2022	97	104
B2	F	39	3 (AAP)	Yes	BA.2	05.03.2022	81	101
B3	F	32	3 (PPP)	Yes	BA.2	02.03.2022	91	100
B4	F	58	3 (AAP)	Yes	BA.2	06.03.2022	105	93
B5	M	27	3 (AAP)	Yes	BA.2	06.03.2022	62	93
B6	M	31	3 (PPP)	Yes	BA.2	07.03.2022	81	92
B7	F	51	3 (AAM)	Yes	BA.2	08.03.2022	119	91
B8	M	32	3 (APP)	Yes	BA.2	10.03.2022	91	89
B9	M	24	3 (AAP)	Yes	BA.2	15.03.2022	115	84
B10	F	52	3 (AAP)	Yes	BA.2	16.03.2022	110	83
B11	F	59	3 (AAP)	Yes	BA.2	18.03.2022	119	81
B12	F	23	3 (AAP)	Yes	BA.2	21.03.2022	131	78
B13	M	28	3 (MMP)	Yes	BA.2	21.03.2022	112	78
B14	F	30	3 (AAP)	Yes	BA.2	22.03.2022	132	77
B15	F	48	3 (AAP)	Yes	BA.2	23.03.2022	126	76
B16	F	28	3 (PPM)	Yes	BA.2	23.03.2022	85	76
B17	M	30	3 (AAP)	Yes	BA.2	25.03.2022	128	74
B18	F	26	3 (AAP)	Yes	BA.2	13.04.2022	135	55
B19	M	23	3 (AAP)	Yes	BA.2	01.05.2022	152	37
B20	M	43	3 (AAP)	Yes	BA.2	08.05.2022	172	30
B21	F	23	3 (AMP)	Yes	BA.2	18.05.2022	169	20
B22	F	29	3 (AAP)	Yes	BA.2	03.06.2022	206	18

n = 22
Age, Geometric mean (CI 95%): 33.4 (4.8)
Sex ratio (M/F): 36% / 64%
Days between 3rd vaccination and infection, Geometric mean (CI 95%): 114.6 (14.2)
Days after infection, Geometric mean (CI 95%): 67.4 (10.7)
*A: ChAdOx1 (AstraZeneca), M: mRNA-1273 (Moderna), P: BNT162 (Biontech Pfizer)

Table S2: Vaccinated and SARS-CoV-2 BA.2 recovered individuals (Vac/BA.2-conv. n=22).

Sex, age, vaccination/convalescent status, days between 3rd immunization and infection as well as days after infection are presented.

Table S3: Vaccinated but non-convalescent individuals (Vac)

ID	Sex	Age	# of vaccinations (scheme*)	COVID19 recovered	Variant	Date of diagnosis	Days after last immunization
C1	F	58	3 (AAP)	No	-	-	312
C2	F	36	3 (PPP)	No	-	-	216
C3	F	29	3 (AAP)	No	-	-	210
C4	M	43	3 (AAP)	No	-	-	209
C5	M	57	3 (AAP)	No	-	-	206
C6	F	35	3 (AAP)	No	-	-	206
C7	M	52	3 (AAP)	No	-	-	204
C8	M	40	3 (AAP)	No	-	-	202
C9	F	39	3 (AAP)	No	-	-	201
C10	F	57	3 (AAM)	No	-	-	201
C11	F	30	3 (PPP)	No	-	-	199
C12	F	46	3 (AAM)	No	-	-	195
C13	F	24	3 (AAP)	No	-	-	193
C14	F	22	3 (PPP)	No	-	-	193
C15	M	29	3 (AAP)	No	-	-	193
C16	F	30	3 (AAP)	No	-	-	193
C17	F	59	3 (AAM)	No	-	-	189
C18	F	57	3 (AAM)	No	-	-	189
C19	F	42	3 (AAM)	No	-	-	189
C20	F	26	3 (AAP)	No	-	-	185
C21	M	36	3 (AAP)	No	-	-	174
C22	F	30	3 (APP)	No	-	-	172
C23	F	31	3 (AAP)	No	-	-	171
C24	M	33	3 (AAP)	No	-	-	143

n = 24
Age, Geometric mean (CI 95%): 37.6 (4.7)
Sex ratio (M/F): 29% / 71%
Days after last immunization, Geometric mean (CI 95%): 196.0 (11.6)
*A: ChAdOx1 (AstraZeneca), M: mRNA-1273 (Moderna), P: BNT162 (Biontech Pfizer)

Table S3: Vaccinated but non-convalescent individuals (Vac. n=24).

Sex, age, vaccination/convalescent status and days after last immunization are presented.

Table S4: Unvaccinated but SARS-CoV-2 BA.1 convalescent individuals (BA.1-conv)

ID	Sex	Age	# of vaccinations (scheme*)	COVID19 recovered	Variant	Date of diagnosis	Days after infection
D1	M	73	-	Yes	BA.1	20.12.2021	171
D2	M	75	-	Yes	BA.1	20.12.2021	171
D3	F	73	-	Yes	BA.1	03.01.2022	157
D4	F	57	-	Yes	BA.1	21.01.2022	137
D5	M	24	-	Yes	BA.1	27.01.2022	137
D6	F	44	-	Yes	BA.1	25.01.2022	133
D7	F	23	-	Yes	BA.1	02.02.2022	131
D8	M	34	-	Yes	BA.1	31.01.2022	127
D9	M	45	-	Yes	BA.1	01.02.2022	127
D10	F	39	-	Yes	BA.1	19.02.2022	110
D11	M	16	-	Yes	BA.1	23.02.2022	106
D12	M	75	-	Yes	BA.1	20.12.2021	81
D13	M	73	-	Yes	BA.1	20.12.2021	81
D14	F	42	-	Yes	BA.1	08.12.2021	79
D15	F	72	-	Yes	BA.1	03.01.2022	67
D16	F	28	-	Yes	BA.1	25.01.2022	31
D17	F	40	-	Yes	BA.1	15.01.2022	27
D18	M	33	-	Yes	BA.1	31.01.2022	25
D19	M	45	-	Yes	BA.1	01.02.2022	24
D20	F	38	-	Yes	BA.1	19.02.2022	20
D21	M	24	-	Yes	BA.1	27.01.2022	18
D22	F	44	-	Yes	BA.1	25.01.2022	16
D23	M	16	-	Yes	BA.1	23.02.2022	16
D24	F	23	-	Yes	BA.1	02.02.2022	12

n = 24
Age, Geometric mean (CI 95%): 39.6 (8.0)
Sex ratio (M/F): 50% / 50%
Days after infection, Geometric mean (CI 95%): 60.3 (22.4)
*A: ChAdOx1 (AstraZeneca), M: mRNA-1273 (Moderna), P: BNT162 (Biontech Pfizer)

Table S4: Unvaccinated but SARS-CoV-2 BA.1 convalescent individuals (BA.1-conv. n=24).

Sex, age, vaccination/convalescent status and days after infection are presented.

Abbreviations

Vac/BA.1-conv	group of BA.1 convalescent patients who were fully vaccinated and received a heterologous or homologous booster dose
Vac/BA.2-conv	group of BA.2 convalescent patients who were fully vaccinated and received a heterologous or homologous booster dose
Vac	group of fully vaccinated who received a heterologous or homologous booster dose
Ab	antibody, immunoglobulin
BA.1	SARS-CoV-2 Omicron BA.1 variant
BA.1-conv	group of BA.1 convalescent patients who were not vaccinated
BA.2	SARS-CoV-2 Omicron BA.2 variant
BA.4/5	SARS-CoV-2 Omicron BA.4 or BA.5 variants
BAU	binding antibody unit
BNT162b2	BioNTech/Pfizer COVID-19 vaccine
ChAdOx1	AstraZeneca COVID-19 vaccine
CI	confidence interval
COVID-19	Coronavirus disease 19
Ig	immunoglobulin, antibody
IgG	immunoglobulin G
mRNA	messenger ribonucleic acid
mRNA-1273	Moderna COVID-19 vaccine
NT_{50}	neutralization titer reduction of 50%
PBMCs	Peripheral blood mononuclear cells
PFU	plaque forming units
RBD	receptor binding domain
S	SARS-CoV-2 spike protein
S1	SARS-CoV-2 spike protein region 1
SARS-CoV-2	severe acute respiratory syndrome coronavirus type 2
VOCs	variants of concern
WT	SARS-CoV-2 wildtype
